# Supplementary material for: Detection of Human p53 In-Vitro Expressed in a Transcription-Translation Cell-Free System by a Novel Conjugate Based on Cadmium Sulphide Nanoparticles
Source: Nanomaterials (Basel). 2020 May 21;10(5):984. doi: 10.3390/nano10050984 (PMC7279493; doi:10.3390/nano10050984)
Supplement: Supplementary file 1 [file nanomaterials-10-00984-s001.pdf]

nanomaterials

MDPI

Electronic Supplementary Material

Detection of human p53 *in-vitro* expressed in a transcription-translation cell-free system by a novel conjugate based on cadmium sulphide nanoparticles

Víctor Barba-Vicente[1], María Jesús Almendral Parra[1*], Juan Francisco Boyero-Benito[1], Carlota Auría-Soro[1,3], Pablo Juanes-Velasco[2,3], Alicia Landeria-Viñuela[2,3], Álvaro Furones-Cuadrado[3], Ángela-Patricia Hernández[3], Raúl Manzano-Román[2] and Manuel Fuentes[2,3*]

1 Department of Analytical Chemistry, Nutrition and Food Science, Faculty of Chemistry, University of Salamanca, 37008 Salamanca, Spain.
2 Proteomics Unit. Cancer Research Centre (IBMCC/CSIC/USAL/IBSAL), Salamanca, Spain.
3 Department of Medicine and Cytometry General Service-Nucleus. CIBERONC CB16/12/00400. Cancer Research Centre (IBMCC/CSIC/USAL/IBSAL), 37007 Salamanca, Spain.

* Co-corresponding authors: Manuel Fuentes, mfuentes@usal.es; María Jesus Almendral, almendral@usal.es Tel.: +34923294811 (M.F.); +34923218526 (MJA).

S1. Calculating the average diameter of the nanoparticles

As from the wavelength of the excitonic peak maximum of its absorption spectrum.

The average diameter of the nanoparticles can be calculated as from the wavelength of the excitonic peak maximum of its absorption spectrum.

One of the consequences of the reduction in size of a semiconductor crystal as it undergoes a quantum confinement is that the bandgap energy is gradually modified towards more energetic values the smaller the size.

The increase in energy of the empty band of the semiconductor is related to the size of the nanoparticle according to expression (1) [1-4]

$$E_{efc} = E^0 + \frac{h^2}{8r^2 m_{er}} - \frac{1.8e^2}{4\pi\varepsilon_0 \varepsilon\, r} \qquad (1)$$

in which E_{efc} is the effective energy of the empty band of the nanocrystal (eV); E^0 is the energy of the empty band of the unitary cell of the crystal; r is the radius of the nanoparticle; h is the Planck constant; m_{er} is the effective reduced mass of the electron; e is the electron charge at rest; and ε_0 and ε are the dielectric constants of the crystal and the nanocrystal respectively.

The last of the terms of the equation is numerically negligible compared with the other two, owing to which the expression (1) for practical calculation purposes is simplified to:

$$E_{efc} = E^0 + \frac{h^2}{8r^2 m_{er}} \quad (2)$$

The effective energy of the empty band of the nanocrystal is calculated as from the wavelength of the excitonic peak maximum (Λ_{max}), as from the E_{efec}= h c/$\Lambda_{max.}$ relation. In the case of the CdS the E_0 is 2.4 eV [5-7] which is equivalent to a wavelength of 515 nm, which in the case of the unitary crystal corresponds to the cutting wavelength of the absorption spectrum (initiation of the absorption). We will call this wavelength $\lambda_{crystal}$.

The effective reduced mass of the electron is related to the effective masses of the electron and of the gap according to the expression:

$$m_{er} = \frac{m_e m_h}{m_e + m_h} \quad (3)$$

in which m_e is the effective mass of the electron and m_h is the effective mass of the gap. In the case of CdS, m_e= 0.19 m_0 and m_h= 0.80 m_0 (with m_0 being the mass of the electron at rest). As from the expression (2) it is possible to obtain the value of r:

$$r = \sqrt{\frac{h^2}{\left(E_{efc} - E^0\right) 8 m_{er}}} \quad (4)$$

As the experimental piece of data obtained from the absorption spectre is Λ_{max}, it is possible to transform the expression (4) into another in accordance with Λ_{max} taking into account that *h*= 6.6252×10^{-27} erg s; 1 eV= 1.602×10^{-12} erg; m_0= 9.1096×10^{-28} g; *c*= 3.0×10^{10} cm s^{-1}; and 1 nm= 10^{-7} cm; $\lambda_{cristal}$= 515 nm; which together with the radius of the nanoparticles and the Λ_{max} gives the following result:

$$r(nm) = 0.0444 \sqrt{\frac{515 \lambda_{max}}{515 - \lambda_{max}}} \quad (5)$$

This last method for calculating the average size of the nanoparticles causes problems as to the exact calculation when the spectrum of the NPs does not have a well defined absorption maximum. In these cases it is more practical to obtain the value of d from the cutting wavelength of the absorption spectrum of the nanoparticles by using the well known empiric expression deduced by Henglein [8], who relates it to the cutting wavelength of the lower part of the spectrum with the baseline (λ_{edge}, λ_e o λ_{corte}) expressed in nm.

$$d = \frac{0.1}{\left(0.138 - 0.0002345\, \lambda_e\right)} \quad (6)$$

S2. Estimating the number of CdS molecules contained in the nanoparticles

When the mean size of the nanoparticles is known it is possible to estimate the number of CdS molecules contained in each QD. This calculation is possible owing to the spherical morphology of the NPs obtained.

As the volume of the sphere is $4/3\pi r^3$, the number of CdS molecules inside each particle can be estimated by means of the expression:

$$N_{CdS} = \frac{4}{3}\pi r^3 \frac{\rho_{CdS} N_0}{M_{CdS}} \qquad (7)$$

in which N_{CdS} is the number of CdS molecules; r is the radius of the nanoparticle; ρ_{CdS} is the CdS density (4.82 g cm^{-3}); N_0 is the Avogadro number (6.023x10^{23} molecules mol^{-1}); and M_{CdS} is the CdS molecular mass (144.46 g mol^{-1}).

In the case of the CdS nanoparticles, the expression (7) which replaces the value of the constants in coherent units is transformed into:

$$N_{CdS} = 81.8\, r^3 \quad (r\ in\ nm) \qquad (8)$$

Therefore, for the nanoparticles responsible for the spectrum in Figure 1 (Original Paper) in which r =1.39 nm, it is obtained as from expression (8) in which N_{CdS}= 220, which gives a clear idea of the quantum confinement by size.

S3. Determining the concentration of nanoparticles in solution and the molar extinction coefficient

The maximum absorbance value is related to the concentration of CdS NPs in solution and also to the ε molar extinction coefficient, which depends on the size of the nanoparticles.

If the solubility product of the CdS (pK_{CdS}= 27) is taken into account and the fact that the pH at which the nanoparticles are obtained (between 9 and 11) is the minimum solubility area of the CdS, we can consider that the concentration of CdS intervening in the formation of nanoparticles is similar to the concentration of limiting reagent that is added.

If we follow this reasoning, the concentration of NPs in solution can be calculated by taking into account the N_{CdS} value deduced as from the mean size of the NP:

$$E_{efc} = E^0 + \frac{h^2}{8r^2 m_{er}} \qquad C_{Cds} = \frac{[CdS]}{N} = \frac{[S^{2-}]}{N} \qquad (9)$$

The value of Ɛ can be deduced for each case from the maximum spectrum absorption value:

$$\varepsilon = \frac{A_{max}}{C_{CdS}} \qquad (10)$$

S4. Effect of the pH of the medium on the synthesis of CDs-BSA QDs

Determining the size of the NPs, the number of CdS molecules per NP, the concentration of NPs, and ε molar absorptivity for the different pH values studied (Tables S1-S4)

Table S1. The obtaining of CdS nanoparticles using thioacetamide as a precursor. Calculating the mean size of the nanoparticles and the number of molecules per nanoparticle at different evolution times. pH=9. $[Cd^{2+}]$ = 7.477x10^{-4} mol/L; [BSA] = 3.738x10^{-4} mol/L; [Thioac.] = 3.738x10^{-4} mol/L; $[Cd^{2+}]_F$/$[Thioac.]_F$ = 2.0; $[Cd^{2+}]_F$/$[BSA]_F$ = 2.0 room temperature.

Time/days	λmax/nm	Diameter/nm	N_{CdS}/molecules	Absorbance /u.a.	C_{CdS}/ mol L^{-1}	ε/ mol^{-1} L cm^{-1}
**0**	-	-	-		-	-
**1**	355.0	3.00	277	0.104	1.35E-06	7.7E+04
**2**	365.0	3.14	318	0.172	1.18E-06	1.5E+05
**3**	365.0	3.14	318	0.194	1.18E-06	1.7E+05
**7**	366.0	3.16	322	0.235	1.16E-06	2.0E+05
**8**	369.0	3.20	336	0.297	1.11E-06	2.7E+05
**9**	372.0	3.26	351	0.318	1.06E-06	3.0E+05
**10**	372.5	3.26	354	0.321	1.06E-06	3.0E+05
**13**	371.5	3.24	349	0.338	1.07E-06	3.2E+05

Table S2. The obtaining of CdS nanoparticles using thioacetamide as a precursor. Calculating the mean size of the nanoparticles and the number of molecules per nanoparticle at different evolution times. pH=10. $[Cd^{2+}]$ = 7.477x10^{-4} mol/L; [BSA] = 3.738x10^{-4} mol/L; [Thioac.] = 3.738x10^{-4} M; $[Cd^{2+}]_F$/$[Thioac.]_F$ = 2.0; $[Cd^{2+}]_F$/$[BSA]_F$ = 2.0 room temperature.

Time/days	λmax/nm	Diameter/nm	N_{CdS}/molecules	Absorbance /u.a.	C_{CdS}/ mol L^{-1}	ε/ mol^{-1} L cm^{-1}
**0**	-	-	-	-	-	-
**1**	335.0	2.74	210	1.008	1.78E-06	5.7E+05
**2**	336.0	2.76	215	1.071	1.74E-06	6.2E+05
**3**	338.5	2.78	220	1.134	1.70E-06	6.7E+05
**7**	336.5	2.76	215	1.258	1.74E-06	7.2E+05

**8**	338.5	2.78	220	1.285	1.70E-06	7.6E+05
**9**	339.0	2.80	224	1.332	1.67E-06	8.0E+05
**10**	339.0	2.80	224	1.328	1.67E-06	8.0E+05
**14**	339.0	2.80	224	1.378	1.67E-06	8.3E+05
**16**	339.0	2.80	224	1.088	1.67E-06	6.5E+05
**21**	339.0	2.80	224	1.084	1.67E-06	6.5E+05
**28**	339.0	2.80	224	1.076	1.67E-06	6.5E+05

Table S3. The obtaining of CdS nanoparticles using thioacetamide as a precursor. Calculating the mean size of the nanoparticles and the number of molecules per nanoparticle at different evolution times. pH=10.5. $[Cd^{2+}]$ = 7.477x10^{-4} mol/L; [BSA] = 3.738x10^{-4} mol/L; [Thioac.] = 3.738x10^{-4} mol/L; $[Cd^{2+}]_F/[Thioac.]_F$ = 2.0; $[Cd^{2+}]_F/[BSA]_F$ = 2.0 room temperature.

**Time/days**	**λmax/nm**	**Diameter/nm**	**N_{CdS}/molecules**	**Absorbance /u.a.**	**C_{CdS}/ mol L^{-1}**	**ε/ mol^{-1} L cm^{-1}**
**0**	-	-	-	-	-	-
**1**	332.0	2.72	204	0.524	1.83E-06	2.9E+05
**2**	332.5	2.72	206	0.546	1.82E-06	3.0E+05
**5**	334.0	2.74	210	1.134	1.78E-06	6.4E+05
**6**	334.0	2.74	210	0.982	1.78E-06	5.5E+05
**7**	334.5	2.74	211	0.872	1.77E-06	5.0E+05

Table S4. The obtaining of CdS nanoparticles using thioacetamide as a precursor. Calculating the mean size of the nanoparticles and the number of molecules per nanoparticle at different evolution times. pH=11. $[Cd^{2+}]$ = 7.477x10^{-4} mol/L; [BSA] = 3.738x10^{-4} mol/L; [Thioac.] = 3.738x10^{-4} mol/L; $[Cd^{2+}]_F/[Thioac.]_F$ = 2.0; $[Cd^{2+}]_F/[BSA]_F$ = 2.0 room temperature.

**Time/days**	**λmax/nm**	**Diameter/nm**	**N_{CdS}/molecules**	**Absorbance /u.a.**	**C_{CdS}/ mol L^{-1}**	**ε/ mol^{-1} L cm^{-1}**
**0**	-	-	-	-	-	-
**1**	332.5	2.72	206	0.616	1.82E-06	3.4E+05
**2**	332.0	2.72	204	0.616	1.83E-06	3.4E+05
**5**	336.0	2.76	215	1.094	1.74E-06	6.3E+05
**6**	335.5	2.76	214	1.092	1.75E-06	6.3E+05
**7**	336.0	2.76	215	1.091	1.74E-06	6.3E+05

S5. evolution of the mean diameter of the nanoparticles for the different pH values studied.

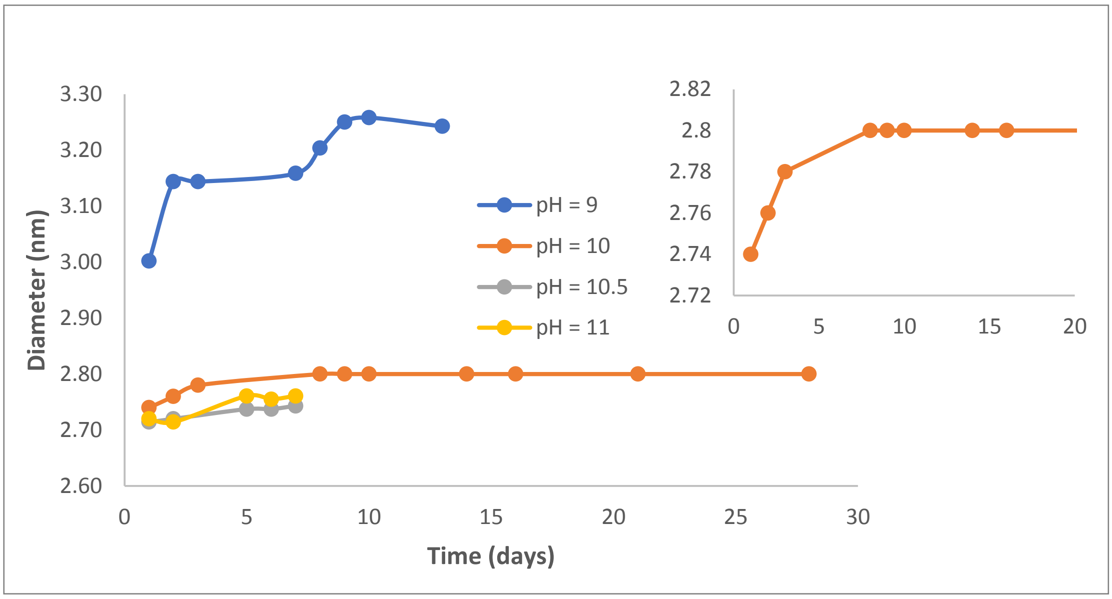

Figure S1. The obtaining of CdS nanoparticles using thioacetamide as a precursor. The evolution of the mean diameter of the nanoparticles at different evolution times for different pHs. $[Cd^{2+}]$ = 7.477x10^{-4} mol/L; [BSA] = 3.738x10^{-4} mol/L; [Thioac.] = 3.738x10^{-4} mol/L; $[Cd^{2+}]_F$/$[Thioac.]_F$ = 2.0; $[Cd^{2+}]_F$/$[BSA]_F$ = 2.0 room temperature.

S6. Effect of the pH of the Medium on the Synthesis of CdS QDs at 4°C

Determining the size of the NPs, the number of CdS molecules per NP, the concentration of NPs, and ε molar absorptivity for the different pH values studied. At 4ºC (Tables S5-S7)

Table S5. The obtaining of CdS nanoparticles using Thioacetamide as a precursor. Calculating the mean size of the nanoparticles and the number of molecules per nanoparticle at different evolution times. pH=9. $[Cd^{2+}]$ = 7.477x10-4 mol/L; [BSA] = 3.738x10-4 mol/L; [thioac.] = 3.738x10-4 M; $[Cd2+]_F$/$[thioac]_F$ = 2.0; $[Cd^{2+}]_F$/$[BSA]_F$ = 2.0. Temperature: 4°C.

Time/days	λmax/nm	Diameter/nm	N_{CdS}/molecules	Absorbance /u.a.	C_{CdS}/ mol L^{-1}	ε/ mol^{-1} L cm^{-1}
**1**	-	-	-	-	-	-
**3**	-	-	-	-	-	-
**13**	-	-	-	-	-	-

**28**	-	-	-	-	-	-
**35**	348.0	2.90	252	0.481	1.48E-06	3.2E+05
**40**	348.0	2.90	252	0.438	1.48E-06	3.0E+05
**56**	348.0	2.90	252	0.438	1.48E-06	3.0E+05

Table S6. The obtaining of CdS nanoparticles using Thioacetamide as a precursor. Calculating the mean size of the nanoparticles and the number of molecules per nanoparticle at different evolution times. pH=10. $[Cd^{2+}]$ = 7.477x10^{-4} mol/L; [BSA] = 3.738x10^{-4} mol/L; [Thioac.] = 3.738x10^{-4} mol/L; $[Cd^{2+}]_F/[Thioac.]_F$= 2.0; $[Cd^{2+}]_F/[BSA]_F$ = 2.0. Temperature: 4°C.

**Time/days**	**λmax/nm**	**Diameter/nm**	**N_{CdS}/molecules**	**Absorbance /u.a.**	**C_{CdS}/ mol L^{-1}**	**Ɛ/ mol^{-1} L cm^{-1}**
**1**	-	-	-	-	-	-
**3**	368.5	3.20	334	0.389	1.12E-06	3.5E+05
**13**	367.5	3.18	329	0.436	1.14E-06	3.8E+05
**28**	372.5	3.26	354	0.423	1.06E-06	4.0E+05
**35**	369.0	3.20	336	0.448	1.11E-06	4.0E+05
**40**	368.0	3.18	331	0.469	1.13E-06	4.2E+05
**69**	379.5	3.38	392	0.404	9.54E-07	4.2E+05

Table S7. The obtaining of CdS nanoparticles using Thioacetamide as a precursor. Calculating the mean size of the nanoparticles and the number of molecules per nanoparticle at different evolution times. pH=11. $[Cd^{2+}]$ = 7.477x10^{-4} mol/L; [BSA] = 3.738x10^{-4} mol/L; [Thioac.] = 3.738x10^{-4} mol/L; $[Cd^{2+}]_F/[Thioac.]_F$ = 2.0; $[Cd^{2+}]_F/[BSA]_F$ = 2.0. Temperature: 4°C.

**Time/days**	**λmax/nm**	**Diameter/nm**	**N_{CdS}/molecules**	**Absorbance /u.a.**	**C_{CdS}/ mol L^{-1}**	**Ɛ/ mol^{-1} L cm^{-1}**
**1**	-	-	-	-	-	-
**3**	359.5	3.06	294	0.470	1.27E-06	3.7E+05
**13**	357.5	3.04	286	0.501	1.31E-06	3.8E+05
**28**	359.5	3.06	294	0.492	1.27E-06	3.9E+05
**35**	357.5	3.04	286	0.493	1.31E-06	3.8E+05
**40**	356.5	3.02	282	0.517	1.33E-06	3.9E+05
**56**	356.5	3.02	282	0.480	1.30E-06	3.7E+05
**69**	361.0	3.08	300	0.396	1.25E-06	3.2E+05
**82**	359.0	3.06	292	0.434	1.28E-06	3.4E+05

S7. Evolution of the mean diameter of the nanoparticles for the different pH values studied.

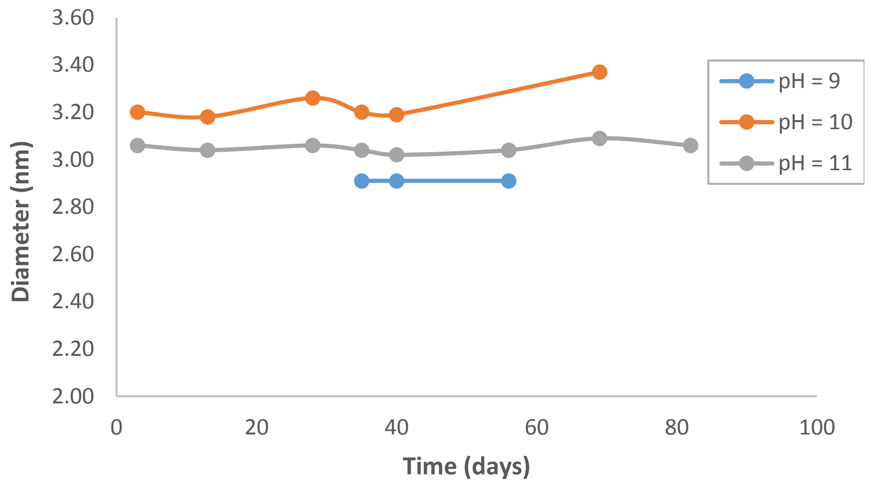

Figure S2. The obtaining of CdS nanoparticles using Thioacetamide as a precursor. The evolution of the mean diameter of the nanoparticles at different times for different pHs. $[Cd^{2+}]$ = 7.477x10^{-4} mol/L; [BSA] = 3.738x10^{-4} mol/L; [Thioac.] = 3.738x10^{-4} mol/L; $[Cd^{2+}]_F$/[Thioac.]$_F$ = 2.0; $[Cd^{2+}]_F$/[BSA]$_F$ = 2.0. Temperature = 4°C.

S8. Emission wavelength values (λ_{em}) and the intensity of the maximum fluorescence ($I_F^{máx}$) of the emission spectra at different times for the nanoparticles synthesized at 4°C, for the different pH values studied.

Table S8. The obtaining of CdS nanoparticles using Thioacetamide as a precursor. Nanoparticles synthesised at an initial pH of 9 and at 4°C.

Time/days	λem/nm	I_F^{max} /u.a.	Δ/nm
0	443.2	320.19	95
2	449.6	312.0.	95
3	448.0	348.86	105
6	457.1	402.11	120
13	468.8	426.68	118
21	475.2	426.68	125
28	476.8	430.78	126
40	488.0	410.30	125

56	488.0	377.53	130

Table S9. The obtaining of CdS nanoparticles using Thioacetamide as a precursor. Nanoparticles synthesised at an initial pH of 10 and at 4ºC.

Time/days	λem/nm	I_F^{max} /u.a.	Δ/nm
0	451.2	312.00	84
2	500.8	942.78	103
3	502.4	987.84	108
6	505.6	1135.02	102
13	507.2	1282.38	100
21	507.2	1319.04	100
28	505.6	1301.65	99
35	504.0	1466.39	99
40	504.0	1314.04	99
69	504.0	1153,53	99

Table S10. The obtaining of CdS nanoparticles using Thioacetamide as a precursor. Nanoparticles synthesized at an initial pH of 11 and at 4ºC.

Time/days	λem/nm	I_F^{max} /u.a.	Δ/nm
0	449.6	299.71	93
2	480.0	627.39	105
3	481.6	783.04	108
6	484.4	1102.46	102
13	483.2	1282.38	103
21	486.4	1666.07	99
28	486.4	1835.23	95
35	488.0	2154.64	97
40	488.0	1947.88	99
56	488.0	2229.68	95
69	486.4	2173.36	96
82	488.0	2286.00	99

S9. Evolution of the diameters of CdS-BSA nanoparticles over time for different concentrations of thioacetamide

Table S11 shows the evolution of the diameters of nanoparticles over time for the different concentrations of thioacetamide studied.

Table S11. The obtaining of CdS nanoparticles using thioacetamide as a precursor. The calculating of the mean diameter of the nanoparticles obtained using different concentrations of thioacetamide. $[Cd^{2+}]$ = 7.477x10^{-4} mol/L; [BSA] = 3.738x10^{-4} mol/L; $[Cd^{2+}]_F/[BSA]_F$ = 2.0. Temperature 4°C.

Days	Diameter			
	Concentration of Thioacetamide			
	1.869x10^{-4} mol/L	3.73810^{-4} mol/L	9.34610^{-6} mol/L	1.869x10^{-3} mol/L
0		2.90	3.00	3.00
1	3.00	3.00	3.10	3.10
4	2.94	3.02	3.10	3.10
7	3.00	3.00	3.10	3.12
36	3.00	3.02	3.06	3.06
78	3.02	3.02	3.08	3.08
82	3.02	3.02	3.08	3.10

S10. Quantum Yield

The fluorescence quantum yield was determined by comparing the integrated emission of the QDs solutions with that of a fluorescent organic molecule (quinine).

$$\Phi_x = \Phi_s\,(K_x/K_s)(\eta_x/\eta_s)^2 \qquad (11)$$

In equation 11, x is the substance to be measured, s is the fluorescence standard, Φ is the quantum yield, η is the refraction index of the solvent used in each solution, and K is the proportionality constant between absorbance and the corrected integrated area of the absorbance spectrum (its ratio should be linear so that the constant K is the slope). Figure S3.

A quantum yield of 22% was found for the QDs solution. Unlike organic fluorophores, the value of Φ in the QDs depends on several variables such as the experimental conditions during their synthesis process, which determine the mean size of the QDs and the greater or lesser capacity of forming the $Cd(OH)_2$ nanoshell, which in turn governs their luminescent properties.

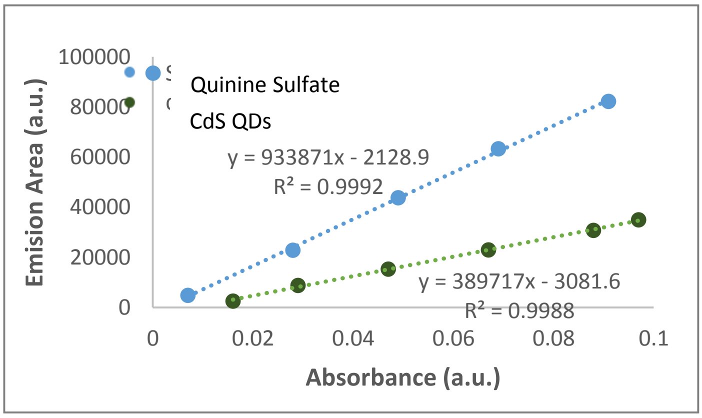

Figure S3. Quantum Efficiency: Representation of the absorbance values compared with the emission area.

S11. Evolution over time of the absorbance values and of the diameters of the NPs synthesised for the different [Cd^{2+}]/[BSA] ratios.

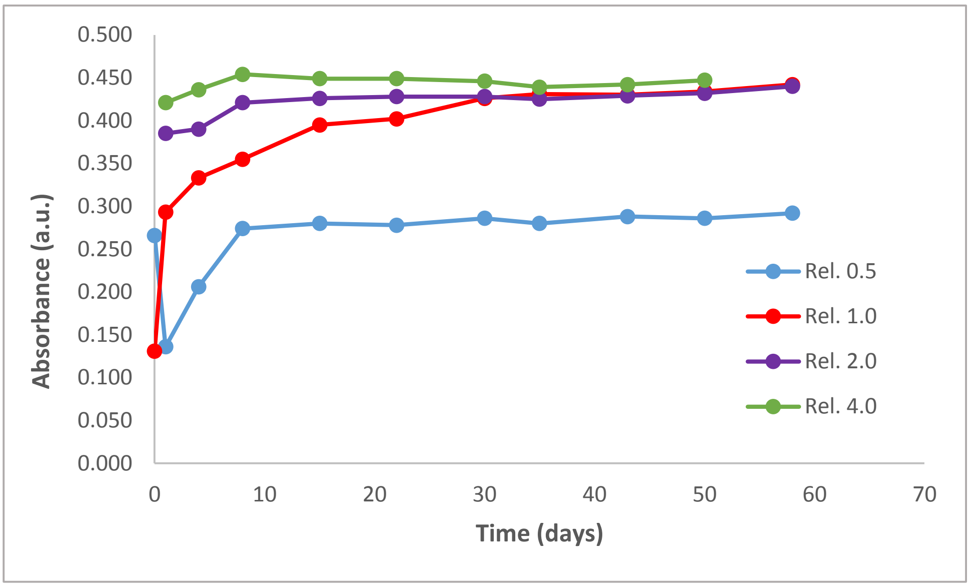

Figure S4. The influence of [Cd^{2+}]$_F$/[BSA]$_F$ at 4°C. The temporal evolution of Absorbance of CdS-BSA QDs solutions with different [Cd^{2+}]$_F$/[BSA]$_F$ ratios.

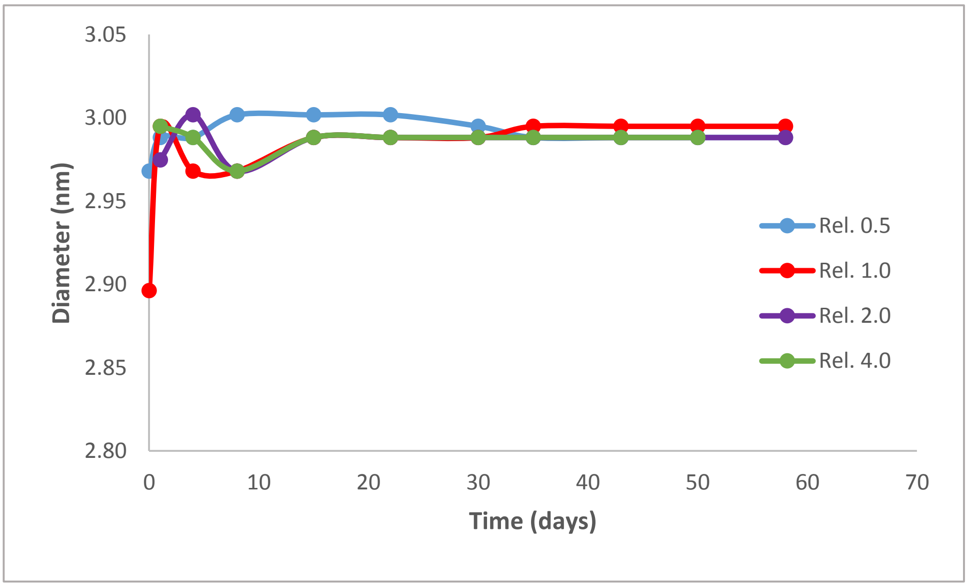

Figure S5. The influence of [Cd^{2+}]$_F$/[BSA]$_F$ ratio at 4°C. The temporal evolution of diameter of CdS-BSA QDs with different [Cd^{2+}]$_F$/[BSA]$_F$ ratios.

S12. Influence of the Amount of QD on the Intensity of Fluorescence Emission

CdS nanoparticles are intended to be used as fluorescent traces in biological media among other applications, taking advantage of their luminescent properties in order to measure an analyte after its chemical bonding to such nanoparticles.

In order to check the proportionality between the concentration of QDs and their fluorescence intensity, a series of solutions was prepared by diluting a solution of QDs stored at 4°C with pH=11. The final concentrations of each species in that solution and their molar ratios were as follows: $[Cd^{2+}]_F$ =7.477x10^{-4} mol/L; $[BSA]_F$ = 3.738x10^{-4} mol/L; $[thioac.]_F$ = 3.738x10^{-4} mol/L; $[Cd^{2+}]_F/[BSA]_F$ = 2.0; $[Cd^{2+}]_F/[thioac.]_F$ = 2.0

6 solutions were prepared from the original one by taking different aliquots of between 0.5 and 8.0 mL. It was found that there is direct proportionality between the concentration of QD and fluorescence emission (y = 246,58x + 9,5713), with a linear regression coefficient of R^2=0.99952 for a 95% confidence interval.

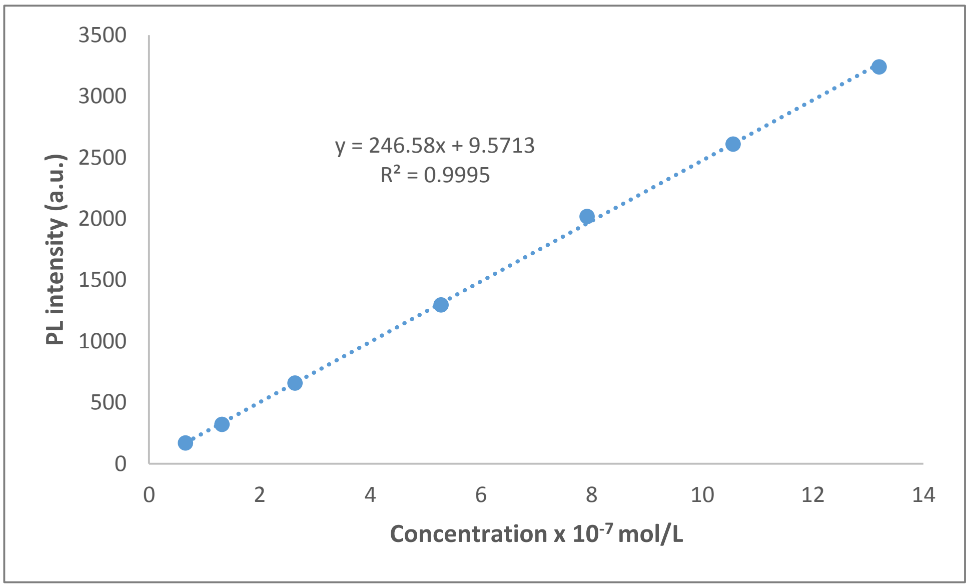

Figure S6. The influence of the Amount of QD on the Intensity of Fluorescence Emission. T= 4°C; pH=11.

The proportionality also shows that although they are diluted the NPs undergo no changes either in structure, size, or surface characteristics. This fact is reaffirmed on checking that the optimum λexc and λem do not vary on carrying out the various dilutions.

S13. Vector employed in IVTT system

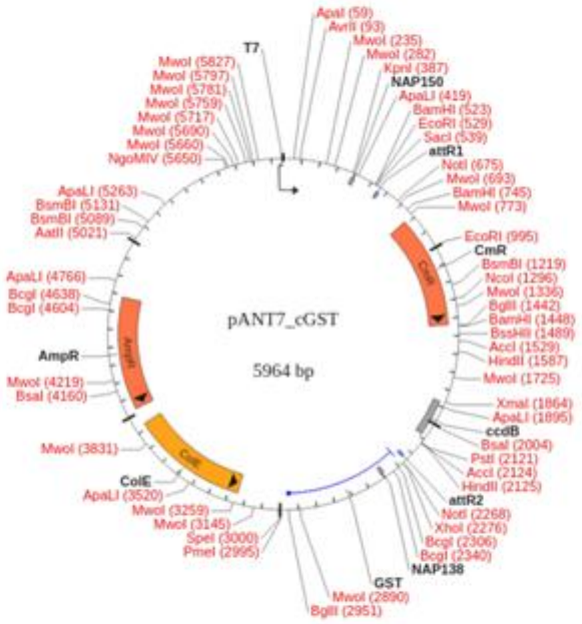

Figure S7. pANT7_cGST map: Schematic representation of vector features for gene insert, T7 polymerase, cloning-subcloning cassettes or resistant cassettes [9].

S14. Analysis IVTT 680 nm

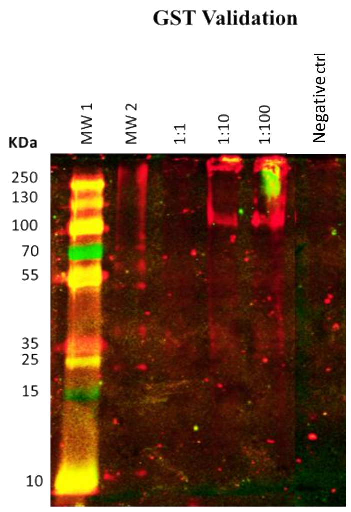

Figure S8: Western Blot analysis of IVTT mix with cDNA 1 µg and 5 µL of IVTT reagents (dilution 1:1 at 1:100 v/v) for the detection of GST at 680 nm.

References

[1] Su H, Han J, Dong Q, Zhang D, Guo Q (2008) In situ synthesis and photoluminescence of QD-CdS on silk fibroin fibers at room temperature. Nanotechnology 19: 025601.

[2] Castanon GAM, Loredo MGS, Mendoza JRM, Ruiz F (2005) Synthesis of CdS nanoparticles: a simple method in aqueous media. Adv. Technol. Mater. Proc. 7: 171-174.

[3] Wang Y, Herron N (1991) Nanometer-Sized Semiconductor Clusters: Materials Synthesis, Quantum Size Effects, and Photophysical Properties. Phys. Chem. 95: 525-532.

[4] Brus LE. (1984) Electron–electron and electron-hole interactions in small semiconductor crystallites: The size dependence of the lowest excited electronic state. J. Chem. Phys. 80: 4403.

[5] Kryukov AI, Kuchmii SY, Pokhodenko VD (2000) Energetics of electron processes in semiconductor photocatalytic systems. Theor. Exp. Chem. 36: 63-81.

[6] S.V. Gaponenko SV (1996) Optical properties of semiconductor nancrystals. Cambridge Univ. Press. Cambridge.

[7] Handbook, Physico-Chemical properties of semiconductor compounds Nauka, Moscow, 1979.

[8] Henglein A (1989) Small-Particle Research: Physicochemical Properties of Extremely Small Colloidal Metal and Semiconductor Particles. Chem. Rev. 89: 1861-1873.

[9] Manzano-Roman R. et al. (2012) Self-assembled Protein Arrays from an Ornithodoros moubata Salivary Gland Expression Library. J. Proteome Res., 11, 12: 5972-5982.
